# The use of hyperpolarised ^13^C-MRI in clinical body imaging to probe cancer metabolism

**DOI:** 10.1038/s41416-020-01224-6

**Published:** 2021-01-28

**Authors:** Ramona Woitek, Ferdia A. Gallagher

**Affiliations:** 1 Department of Radiology, University of Cambridge, Cambridge, UK; 2 Department of Biomedical Imaging and Image-guided Therapy, Medical University of Vienna, Vienna, Austria; 3 Cancer Research UK Cambridge Centre, Cambridge, UK

**Keywords:** Cancer imaging, Cancer metabolism

## Abstract

Metabolic reprogramming is one of the hallmarks of cancer and includes the Warburg effect, which is exhibited by many tumours. This can be exploited by positron emission tomography (PET) as part of routine clinical cancer imaging. However, an emerging and alternative method to detect altered metabolism is carbon-13 magnetic resonance imaging (MRI) following injection of hyperpolarised [1-^13^C]pyruvate. The technique increases the signal-to-noise ratio for the detection of hyperpolarised ^13^C-labelled metabolites by several orders of magnitude and facilitates the dynamic, noninvasive imaging of the exchange of ^13^C-pyruvate to ^13^C-lactate over time. The method has produced promising preclinical results in the area of oncology and is currently being explored in human imaging studies. The first translational studies have demonstrated the safety and feasibility of the technique in patients with prostate, renal, breast and pancreatic cancer, as well as revealing a successful response to treatment in breast and prostate cancer patients at an earlier stage than multiparametric MRI. This review will focus on the strengths of the technique and its applications in the area of oncological body MRI including noninvasive characterisation of disease aggressiveness, mapping of tumour heterogeneity, and early response assessment. A comparison of hyperpolarised ^13^C-MRI with state-of-the-art multiparametric MRI is likely to reveal the unique additional information and applications offered by the technique.

## Background

**Fig. 1:**
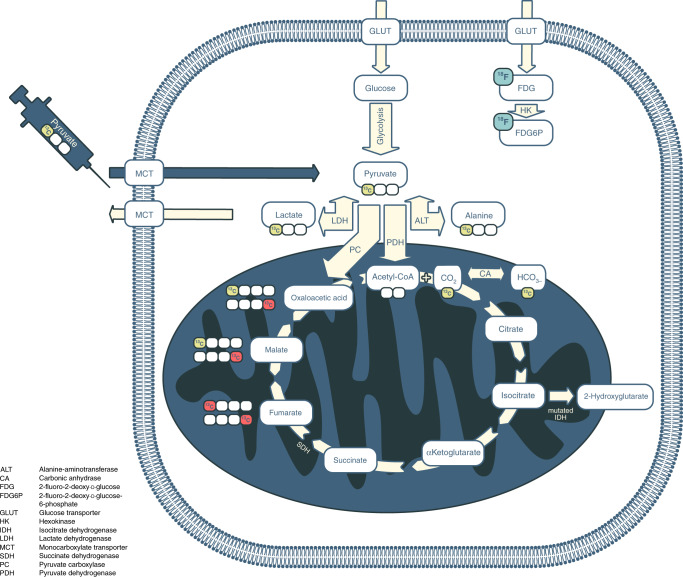
Glucose metabolism in cancer and its relevance for metabolic imaging. Cancer cells frequently demonstrate increased levels of glycolysis including conversion of glucose into pyruvate and subsequently into lactate (the Warburg effect). Consequently, increased import of glucose into tumour cells is maintained by increased expression of glucose transporters (GLUT). The intravenously injected positron emission tomography (PET) tracer [^18^F]2-fluoro-2-deoxy-d-glucose ([^18^F]FDG) is similarly taken up via the same transporter and also undergoes subsequent phosphorylation catalysed by hexokinase; this phosphorylation prevents its subsequent export from the cell. Hyperpolarised [1-^13^C]pyruvate can be used to image metabolic alterations further down the glycolytic pathway. Monocarboxylate transporters (MCTs) mediate its uptake into cancer cells, where it undergoes reduction to [1-^13^C]lactate catalysed by lactate dehydrogenase (LDH), transamination to [1-^13^C]alanine by alanine-aminotransferase (ALT), or irreversible oxidative decarboxylation to acetyl Co-A, a reaction catalysed by pyruvate dehydrogenase (PDH). During the latter oxidation, the hyperpolarised ^13^C-label is transferred from the carboxyl (C1) position of pyruvate to ^13^CO_2_ and is detectable on spectroscopic imaging as bicarbonate (H^13^CO_3_^−^). An alternative fate of [1-^13^C]pyruvate is carboxylation via pyruvate carboxylase (PC) to [1-^13^C]oxaloacetic acid, which can then be metabolised to [1-^13^C]malate. Any fumarate that is formed through the reverse fumarase reaction is symmetrical and therefore any subsequent forward exchange via fumarase results in the production of four-carbon intermediates with the ^13^C-labelling also present at C4 ([4-^13^C]malate and [4-^13^C]oxalacetic acid). The metabolites of this pathway have been detected using hyperpolarised ^13^C-MRI in preclinical liver studies;^110,111^ increased conversion of pyruvate into oxaloacetic acid has been demonstrated in lung and breast cancer using non-imaging studies, which raises important applications for detecting this reaction more generally within tumours.^112^ However, none of these four-carbon intermediates have been detected in cancer using clinical hyperpolarised ^13^C-MRI. The ^13^C-label in the C1 position is shown as yellow circles and in the C4 position as red circles. For clarity, enzymatic cofactors and some of the additional substrates and products have been omitted. Other metabolic alterations relevant for cancer imaging are accumulation of 2-hydroxyglutarate (2HG) due to mutated isocitrate dehydrogenase (IDH) in the tricarboxylic acid cycle and accumulation of succinate due to succinate dehydrogenase (SDH) deficiency.

Metabolic reprogramming is a significant hallmark of most cancers. The Warburg effect is an example of this, using aerobic glycolysis instead of oxidative phosphorylation to produce ATP, and can be demonstrated visually in many cancer patients by the increased uptake of the glucose analogue [^18^F]2-fluoro-2-deoxy-d-glucose using positron emission tomography ([^18^F]FDG-PET) (Fig. 1).^1,2^ However, [^18^F]FDG-PET cannot distinguish the individual metabolites produced from the breakdown of glucose, or their cellular compartmentalisation, and provides no direct information on glycolytic flux or mitochondrial oxidative metabolism. In addition, the ionising radiation emitted during PET poses a small potential risk to patients, which is compounded by [^18^F]FDG-PET typically being performed in conjunction with computed tomography (CT) for localisation and quantification purposes as a hybrid imaging test: the effective dose of a combined PET/CT is ~11 mSv (range 8–24 mSv; ref. ^3^), which equates to approximately five times the average annual exposure from natural background radiation (~2.3 mSv; refs. ^4,5^). The increased risk of both cancer and heritable effects due to ionising radiation is ~5% per Sievert (Sv), which is equivalent to ~0.05% for a PET/CT.^6^ Another limitation of the technique is that [^18^F]FDG-PET demonstrates low sensitivity in a number of cases: in tumours with limited [^18^F]FDG uptake, such as prostate cancer;^7^ in tumours with adjacent high physiological [^18^F]FDG uptake, such as brain tumours surrounded by normal brain tissue; or in cancer of the prostate neighbouring the bladder.^8,9^ Furthermore, increased [^18^F]FDG uptake due to infection or inflammation limits the specificity of the technique but can also be seen as a result of immune infiltration in tumours responding to treatment, thus limiting the accuracy of the technique for early response assessment.^10,11^

In contrast with PET, magnetic resonance imaging (MRI) is a nonionising technique that is used for studying the structure and function of soft tissues, including tumours.^12^ Systems that integrate PET with MRI are increasingly being used for clinical body imaging in oncology to correlate metabolic findings with other functional and structural features. PET-MRI has applications in body regions where MRI is superior to CT for demonstrating soft-tissue contrast, such as in rectal, prostate, hepatobiliary and gynaecological cancers, as well as in brain tumours. Advances in attenuation correction and motion correction have helped to establish PET-MRI as a hybrid imaging technology in clinical oncology.^13^ Furthermore, preclinical data show that a differential response in pyruvate metabolism and [^18^F]FDG uptake might exist following treatment in some tumour models, suggesting that MRI and PET could have complementary roles that could be exploited in the setting of combined PET-MRI.^14^

One of the major strengths afforded by MRI is the ability to noninvasively detect and probe the levels of many tissue metabolites simultaneously and distinguish between them using MR spectroscopy (MRS): certain nuclei resonate in a magnetic field at a frequency that is determined by the local chemical environment. MRS using the proton resonance of hydrogen (^1^H) atoms can facilitate the noninvasive detection, identification and quantification of numerous biochemical compounds or metabolites, such as choline, lactate and many others. Some examples of the use of ^1^H-MRS for detecting phospholipid metabolism, glycolysis, cellular energetics and altered mitochondrial function in tumours are provided in Box 1. However, the use of ^1^H-MRS in routine clinical practice is largely limited to specific applications in neuroradiology and prostate cancer, in part due to in the requirements for specialist data acquisition and analysis methods, which are not widely available.^32,33^

Carbon-13 (^13^C), a nonradioactive form of carbon found at a natural abundance of 1.1%, can also be detected with MRS. As carbon forms the backbone of many endogenous metabolites, ^13^C-MRS offers the possibility of noninvasively detecting and probing many metabolic reactions in vivo. However, conventional ^13^C-MRS is limited by the spatial and temporal resolution that can be achieved, particularly in the clinical setting. In this review, we outline the role for hyperpolarised ^13^C-MRI, an emerging technique that increases the signal-to-noise ratio for the detection of hyperpolarised ^13^C-labelled metabolites by several orders of magnitude, focusing on its applications in the area of oncological body MRI including noninvasive characterisation of disease aggressiveness, mapping of tumour heterogeneity and early response assessment.

Box 1 Tumours frequently display altered phospholipid metabolism, which can be detected using proton (^1^H) MRS in the form of a prominent signal from choline-containing compounds that are involved in phospholipid metabolism and therefore the cellular membranes that form during tumour proliferation.^15–17^ In breast cancer, for example, high levels of choline-containing molecules involved in phospholipid metabolism result in an increased total choline (tCho) signal, which consists of signals from phosphocholine (PC), glycerophosphocholine (GPC) and free choline.^18,19^ An increased tCho signal can be observed in malignant breast lesions but not in benign lesions or in normal breast tissue, and has been shown to decrease during neoadjuvant treatment.^20–22^

As a result of the shift towards increased glycolysis and lactate formation in cancers due to the Warburg effect, an elevated lactate signal on ^1^H-MRS is an indicator of advanced tumour grade and can distinguish cancer recurrence from treatment-related changes.^23,24^ However, in breast cancer, high levels of fat in the peritumoural mammary tissue make lactate detection using ^1^H-MRS extremely challenging and this has limited the application of the technique in routine patient care.^25^

^1^H-MRS has been shown to differentiate prostate adenocarcinoma from normal tissue using the higher (choline + creatine)/citrate ratio in cancer.^26^ Metabolites under the creatine peak (Cr) contribute to energy metabolism while citrate is found at high abundance in normal prostate tissue.^27^ However, despite this, ^1^H-MRS does not feature in the current Prostate Imaging-Reporting and Data System (PI-RADS v 2.1), although its use in the future has not been precluded.^28^

The discoveries of metabolic mutations in some cancers, coupled with improvements in image/data acquisition, have allowed ^1^H-MRS to be applied to the specific detection of tumour-promoting metabolites or oncometabolites. In body imaging, this approach has been applied to the detection of the accumulation of intracellular succinate from succinate dehydrogenase (SDH)-deficient tumours including gastrointestinal stromal tumours, paragangliomas and phaeochromocytomas.^29,30^ Changes in succinate concentration over time have also been demonstrated in patients undergoing therapy.^29,30^ Moreover, ^1^H-MRS has been used to detect the accumulation of fumarate in patients with hereditary leiomyomatosis and renal cell carcinoma, who harbour mutations in the fumarate hydratase gene.^31^

## Hyperpolarised MRI and pyruvate metabolism

Tumour metabolites, such as choline, lactate, succinate and fumarate, can be detected using ^1^H-MRS because they often give rise to discrete peaks that are present at high concentration, often in the millimolar range. However, metabolites that occur at low concentrations, or which generate overlapping resonance peaks on ^1^H-MRS, are more difficult to discriminate. By contrast, metabolite peaks detected using ^13^C-MRS are easier to separate as they are more widely dispersed. The sensitivity for detecting the many metabolic reactions that involve ^13^C can be significantly enhanced by the oral or intravenous administration of ^13^C-enriched compounds, and this approach has been applied to both preclinical and clinical research to interrogate a large range of metabolic pathways. Glycolysis, the tricarboxylic acid (TCA) cycle, as well as pathways intersecting with these two have been the most widely studied.^34^

Hyperpolarisation of ^13^C is an emerging method that increases the signal-to-noise ratio by greater than 10^4^-fold over ^13^C signal acquired at thermal equilibrium (i.e. nonhyperpolarised).^35^ The technique of hyperpolarisation involves increasing the nuclear polarisation of a molecule, which consequently increases the available signal that can be acquired using MR. Although polarisation levels for ^13^C-pyruvate of as high as 70% have been reported using experimental systems, current clinical hyperpolarisers typically polarise molecules of biological interest, such as pyruvate, in the range of 20–30% in the liquid state, which equates to a 10^5^-fold signal enhancement when ^13^C is imaged at 3 Tesla (T), compared with its thermodynamic polarisation at the same field strength.^36,37^

### The hyperpolarisation process

**Fig. 2:**
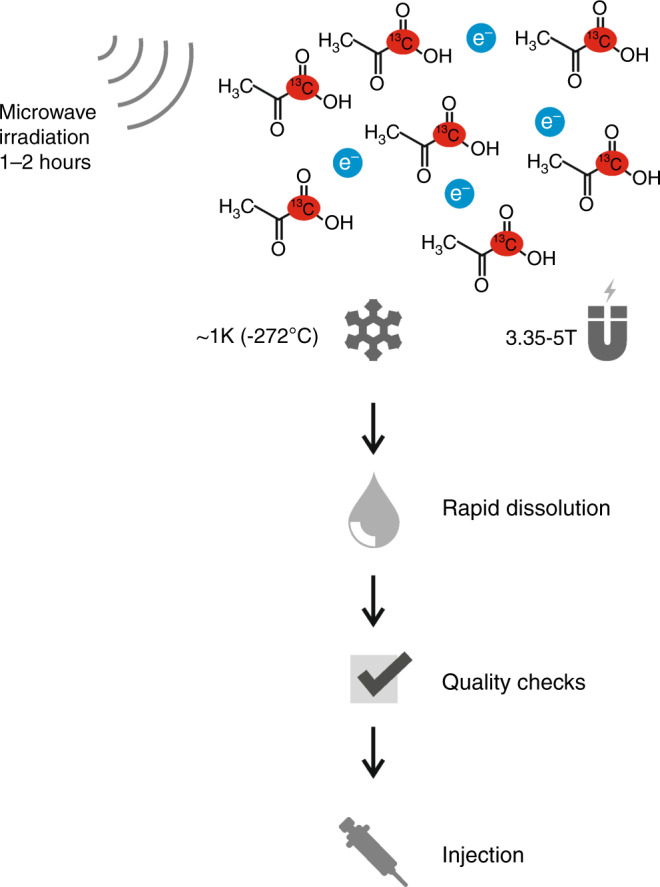
Schematic of the hyperpolarisation process. A solution of ^13^C-labelled pyruvate is doped with a free radical (electrons show in blue) to facilitate hyperpolarisation, while placed in a magnetic field of around 3.35–5 T, cooled to ~1 K (–272 °C), and irradiated with microwaves. After rapid dissolution and successful quality checks (temperature, pH etc.), the solution can be injected intravenously.

Hyperpolarisation utilises the principles of dynamic nuclear polarisation (DNP) to polarise a frozen solution in the solid state with subsequent rapid dissolution while preserving polarisation during the transition into the liquid state for eventual injection into patients. The process is shown by the schematic in Fig. 2: at the beginning of the process, endogenous metabolites labelled with ^13^C are doped with a free radical (electron paramagnetic agent, EPA) to facilitate polarisation by providing unpaired electrons, placed in a magnetic field (typically 3.35–5 T) and cooled to ~1 K (−272 °C). Microwave irradiation at frequencies close to the resonant frequency of the electron spin over 1–2 h is then used to transfer spin polarisation from free electrons to the ^13^C nuclei in the solid state. Subsequently, the sample is rapidly dissolved using superheated water and the radical is removed for patient studies. After having passed suitability checks for use in patients (e.g. pyruvate concentration, pH, residual EPA concentration, and temperature), the solution containing the hyperpolarised ^13^C-labelled molecule is then injected intravenously as quickly as can be technically and safely achieved (usually within a minute) to minimise polarisation loss due to the relatively rapid decay of signal as a result of the short lifetime of the hyperpolarised signal (which decays exponentially with a time constant termed T_1_). Rapid quality checks, injection, and data acquisition are essential to allow insight into metabolic exchange rates and enzymatic activity, as only ~5% of the initial ^13^C signal remains at three T_1_s and <1% at five T_1s._^37^

### Hyperpolarised ^13^C-pyruvate as a probe

Hyperpolarised ^13^C is most often incorporated into pyruvate for preclinical and clinical imaging, as it is an endogenous molecule with a number of favourable biological, physical and chemical properties. Pyruvate is a breakdown product of glucose and can be used to probe several important cellular pathways, as it occupies a key position in cell metabolism at the branch point between reduction to lactate in the cytosol and intramitochondrial oxidative phosphorylation in the citric acid cycle. The low natural abundance of ^13^C (1.1%), the gyromagnetic ratio of ^13^C being a quarter that of ^1^H, and the low endogenous concentration of pyruvate in tissue all mean that detection of the natural abundance of ^13^C-pyruvate using ^13^C-MRI is not feasible in a clinical setting—hyperpolarised ^13^C-pyruvate therefore offers a potential solution to this problem. Pyruvate labelled with ^13^C in the carboxyl position ([1-^13^C]pyruvate) is most frequently used in vivo for hyperpolarised ^13^C-MRI, as the time constant for loss of the hyperpolarised signal (T_1_) is long at clinical field strengths and enables metabolism to be studied over 1–2 min: the T_1_ of [1-^13^C]pyruvate is ~60 seconds ex vivo and ~25–30 seconds in vivo.^38^ Furthermore, low toxicity, rapid intracellular uptake and fast conversion into observable metabolites with a significant chemical shift difference, such as lactate, bicarbonate and alanine, make pyruvate an ideal molecule for clinical use.

### Uses of hyperpolarised ^13^C-pyruvate

Hyperpolarised ^13^C-pyruvate is injected into patients intravenously at a concentration of 250 mM, which produces an approximate intravascular concentration of ~1–2 mM and a tissue concentration of ~0.1 mM—similar to the physiological concentrations of endogenous pyruvate.^39,40^ Following transport to the tissue of interest, hyperpolarised ^13^C-pyruvate undergoes rapid intracellular uptake mediated by monocarboxylate transporters (MCTs). Transmembrane transport has been identified in preclinical studies to be rate-limiting for the conversion of extracellular pyruvate into lactate, and the importance of MCT1 is supported by the first clinical studies using hyperpolarised ^13^C-MRI in breast and prostate cancer.^41–43^

The intracellular metabolic fate of pyruvate is outlined in Fig. 1: the enzyme lactate dehydrogenase (LDH) catalyses the exchange of hyperpolarised ^13^C-pyruvate with existing endogenous intracellular lactate, and it is this reaction that will form the focus of this review. The underlying biological mechanisms that determine the detection of the hyperpolarised ^13^C-lactate signal vary between tumours, and the measured hyperpolarised lactate signal might depend on a variety of factors, including tissue perfusion, membrane transport of pyruvate (MCT1) and enzymatic activity (LDH), as well as the concentration of both the cofactor for the reaction (NADH) and the product, lactate. For example, if the pool of endogenous lactate increases, the hyperpolarised ^13^C-lactate signal will also increase due to increased ^13^C-label exchange between pyruvate and the elevated lactate pool, as has been demonstrated in many models in vitro.^44^

The amino acid alanine, also an end-product of glucose metabolism, is required for protein synthesis and amino acid transformations, and can also be detected following the injection of hyperpolarised ^13^C-pyruvate. Alanine-aminotransferase (ALT) catalyses the exchange of pyruvate and glutamate to alanine and α-ketoglutarate. Hyperpolarised ^13^C-pyruvate has been shown to label the endogenous alanine pool in normal muscle, liver and kidney.^45–47^ Although the endogenous concentration of alanine is lower in most tumours than in muscle, the formation of hyperpolarised ^13^C-alanine has been demonstrated in several preclinical models of cancer, including pancreatic and prostate cancer.^48,49^ Hyperpolarised ^13^C-alanine has also been detected in human pancreatic adenocarcinoma (PDAC).^50^

A third major reaction that can be detected following injection of hyperpolarised ^13^C-pyruvate is the irreversible formation of hyperpolarised ^13^C-carbon dioxide catalysed by the enzyme pyruvate dehydrogenase (PDH), which controls pyruvate flux into the mitochondria. The ^13^C-carbon dioxide subsequently exchanges with ^13^C-bicarbonate through the action of the enzyme carbonic anhydrase (CA) and, given the greater abundance of bicarbonate compared with carbon dioxide at physiological pH, it is the detection of the former that is used to demonstrate significant PDH activity in vivo. While significant levels of hyperpolarised ^13^C-bicarbonate can be detected in the brain and the heart following injection of hyperpolarised ^13^C-pyruvate,^51,52^ very little PDH activity exists in most tumours due to a reduction in oxidative phosphorylation. However, bicarbonate formation has been demonstrated in some glycolytic glioblastoma models.^53^

## Image acquisition and analysis of hyperpolarised ^13^C-MRI data

The acquisition techniques used for the first study of metabolic imaging using hyperpolarised [1-^13^C]pyruvate in human cancer were mainly 1D and 2D dynamic MRI at a temporal resolution of 3 seconds using echo-planar spectroscopic imaging (EPSI) to establish the time course of ^13^C-pyruvate delivery, and 3D EPSI single timepoint acquisitions in subsequent patients with a small field of view (FOV) of 8–10 cm covering the prostate.^38^ Initial oncological case reports used dynamic ^13^C chemical shift imaging (CSI) in a single slice every 10 seconds in a patient with breast cancer^54^ and every 20 seconds for renal cell cancer,^55^ while 1D dynamic MRI and 2D single timepoint CSI were acquired in a patient with pancreatic cancer.^50^ The first study investigating hyperpolarised ^13^C-MRI in different types of breast cancer and early response assessment in triple-negative breast cancer (TNBC) used IDEAL spiral CSI at a temporal resolution of 4 seconds for dynamic metabolic imaging.^43,56^ Spectral-spatial excitation is increasingly being used in clinical hyperpolarised ^13^C-MRI of the prostate and brain as it increases the signal-to-noise ratio for downstream metabolites that are less abundant than pyruvate, while preserving polarisation of the ^13^C-pyruvate, and can be combined with echo-planar or spiral k-space trajectories.^57–59^

Although the analysis of hyperpolarised ^13^C-MRS or MRI data acquired at single time points can be accomplished by calculating the ^13^C-lactate-to-^13^C-pyruvate ratio, or by dividing ^13^C-lactate by the total signal from all ^13^C metabolites, this approach does not take advantage of the dynamic nature of the data generated in temporally resolved ^13^C studies. Dynamic hyperpolarised ^13^C-MRI confers the potential to provide absolute quantification of metabolic conversion, regardless of differences in bolus delivery or polarisation level, by using kinetic modelling to estimate a pyruvate-to-lactate conversion rate constant (or *k*_PL_) or by using computationally simple approaches, such as the area-under-curve (AUC) ratio between ^13^C-lactate and ^13^C-pyruvate or ^13^C-lactate time-to-peak (TTP).^60–62^ Although the majority of modelling approaches require the arterial input of pyruvate to be modelled using an arterial input function, and the time constant for the loss of the hyperpolarised metabolite signal (T_1_) to be predefined or fitted, an inputless *k*_PL_ fitting method offers the advantage of not requiring this input function and fits only one or two additional variables, which simplifies the analysis. For example, the lactate magnetisation can be fitted (and optionally the T_1_ of lactate, T_1*L*_, which can also be approximated to that of pyruvate), with the measured pyruvate magnetisation being used as the input for the kinetic model at each timepoint.^51,61,63^ The inputless method, with its reduced number of fitted parameters, has been shown to be the most robust approach for data where the signal-to-noise is low or where there are different bolus characteristics using both simulated and real data of human prostate cancer, and is therefore very translatable to the real challenges of many clinical studies.^61^

Consistency in the technical acquisition of data at and between sites is key to the development of the field. Although the development of faster imaging methods that increase the signal-to-noise ratio is important for future clinical applications, these new methods will make comparison between data acquired over time difficult, as they will compare methods acquired over different developmental generations. Furthermore, injection rates can vary between institutions, adding further heterogeneity; indeed, consensus between sites regarding technical acquisition and analysis would be of great value to this field of research. Very few published examples of repeatability studies exist^42^ and more extensive repeatability and reproducibility research needs to be undertaken.

In conclusion, multicentre studies with homogeneous data acquisition protocols and analysis pipelines whose protocols include repeatability and reproducibility studies will be invaluable for the success of this emerging technique and should be a key priority for groups and centres focusing their research on hyperpolarised ^13^C-MRI.

## Early-stage human data and implications for clinical translation from preclinical studies

The technique of hyperpolarised ^13^C-MRI has been applied to a range of preclinical cancer models over the past 15 years.^14,49,59,64–68^ The evidence from these studies overwhelmingly indicates an increased exchange of the hyperpolarised ^13^C-label between the injected pyruvate and the endogenous lactate pool in most cancers that occurs secondary to increased glycolysis as a result of metabolic reprogramming.^66^ Indeed, several studies have shown positive correlations between increased lactate flux on ^13^C-MRI and tumour grade and progression in animal models, such as in mouse models of prostate cancer,^48^ in models of pancreatic preneoplasia/cancer,^49^ and in the early detection of *Myc*-driven liver tumours.^66^ Two clinical studies have shown that increased lactate labelling correlates with increased tumour Gleason grade in prostate cancer and with higher grade breast cancer, including triple-negative breast cancer.^42,43^

A second major potential clinical application of hyperpolarised ^13^C-MRI is the early detection of a successful response to chemotherapy: a reduction in lactate labelling following treatment has been demonstrated across a wide range of tumour models using both chemotherapy and radiotherapy. Early metabolic changes seen using hyperpolarised ^13^C-MRI have been demonstrated between 16 and 48 h after treatment initiation in animal models of breast cancer, colon cancer and lymphoma^14,44,67^ and in patient-derived xenografts of renal cell cancer.^68^ In some models, the method has been shown to be more sensitive than [^18^F]FDG-PET to early-treatment-related changes.^14,68^ Preliminary evidence exists for a reduction in lactate labelling in conjunction with a successful response to treatment in human prostate cancer, following androgen ablation and chemotherapy and in breast cancer after a single cycle of neoadjuvant chemotherapy.^56,69^

Although the majority of preclinical models have shown a reduction in lactate labelling following a successful response to treatment, the changes in ^13^C-label exchange between pyruvate and lactate might depend on the type of treatment and combination of drugs. For example, antiangiogenic drugs might produce paradoxical effects because of changes in the delivery of pyruvate and increased hypoxia, which will elevate lactate, and drugs that affect the availability of NAD(H) will have secondary effects on LDH, given that it is a cofactor for this enzyme.^44,70^ Although the preclinical results and early-stage human data appear promising, the use of hyperpolarised ^13^C-MRI in routine clinical care and clinical trials will need to be assessed using larger patient studies. Below, we describe the current status of the field and outline the clinical applications in which hyperpolarised ^13^C-MRI has the potential to inform clinical decision making.

## Potential clinical applications of hyperpolarised ^13^C-MRI

The discussion below focuses on the potential clinical applications of hyperpolarised ^13^C-MRI in oncological body imaging, particularly in prostate, renal, breast and pancreatic cancer. The role of the technique in neurological and cardiological imaging has been reviewed elsewhere^39,71^ and so will not be discussed here.

### Prostate cancer

Although the widespread use of serum prostate-specific antigen (PSA) screening has led to a decrease in cancer-related mortality, there is increasing concern about overdiagnosis and overtreatment of clinically insignificant prostate cancer that has a low risk of progression during the natural lifetime of the patient.^72^ One of the main unmet clinical needs in localised prostate cancer therefore is the stratification of patients in order to receive the most appropriate form of management based on the assessment of tumour aggressiveness e.g. active surveillance (i.e. regular monitoring until treatment is required) or more interventional approaches, such as radical radiotherapy and prostatectomy alone or in combination with antiandrogen therapy and potentially chemotherapy.^73^

Several approaches currently exist in routine clinical practice for predicting prostate tumour aggressiveness. For example, multiparametric MRI (mpMRI) plays an important role in ruling out high Gleason grade disease before treatment and in monitoring the disease by active surveillance.^74,75^ However, despite advances in conventional mpMRI, there is a need to improve both the sensitivity and specificity of the technique for tumour detection. The sensitivity and specificity can vary widely from 53% to 100% and 32% to 97%, respectively; specificity can be lower than 50% for indeterminate radiological lesions.^76–79^ For example, a meta-analysis reported the specificity of mpMRI and prostate imaging-reporting and data system (PI-RADS version 2) for the detection of clinically significant prostate cancer as 62%, when using a cut-off score of ≥4 on PI-RADS, and as low as 29% when using a PI-RADS score of ≥3 (with sensitivities of 90% and 96%, respectively).^78^ When reported on a per-lesion basis, sensitivities tended to be lower, and ranged between 75% and 85%.^80,81^ Therefore, new approaches are required to improve the accuracy of disease characterisation. Assessing tumour metabolism might increase the accuracy with which the disease is classified as clinically significant, and ^1^H-MRS has been used to study tumour metabolism as part of a multiparametric approach, with increased choline and decreased citrate detected in more aggressive tumours. However, ^1^H-MRS is rarely used clinically and its importance has been de-emphasised in the most up-to-date version of PI-RADS.^82^ The use of [^18^F]FDG-PET is limited for the assessment of prostate cancer due to low differential uptake in cancer compared with the normal gland, and the detection of any accumulation in the prostate is often contaminated by high levels of tracer in the adjacent urethra and bladder. In the case of biochemical recurrence (or elevated PSA levels after curative radical prostatectomy), imaging is used to detect and locate early recurrence with the aim of providing curative treatment. mpMRI and targeted PET tracers, such as ^18^F-fluciclovine and ^68^Ga-labelled prostate-specific membrane antigen (PSMA), are currently being investigated for their potential in detecting early recurrence.^83^

Hyperpolarised ^13^C-MRI in patients with prostate cancer has several potential roles, including: improved patient stratification by using ^13^C-lactate labelling to assess aggressiveness; improving diagnostic accuracy for the prediction of disease progression in patients undergoing active surveillance; and locating disease in the case of biochemical recurrence.^84,85^

In the seminal first-in-human study of the technology, 31 patients with prostate cancer were successfully injected with hyperpolarised ^13^C-pyruvate to assess the safety and feasibility of the method.^38^ The results confirmed the preclinical findings of an increased lactate signal-to-noise ratio (SNR) and an elevated apparent exchange rate constant for the enzyme LDH (*k*_PL_) in regions of confirmed prostate cancer, compared with the noncancerous normal gland. In addition, the study provided preliminary evidence that occult low-grade tumours that were not visible on conventional proton mpMRI could be detected by elevated ^13^C-lactate labelling, indicating potentially increased sensitivity also for the detection of recurrence when compared with conventional proton mpMRI. Granlund et al. reported in 2019 that increased lactate labelling correlates with increased tumour Gleason grade in prostate cancer, a finding that suggests a potential application of ^13^C-MRI for the prediction of disease progression in patients undergoing active surveillance.^42^ ^13^C-MRI acquired following injection of hyperpolarised [1-^13^C]pyruvate in twelve patients with prostate cancer demonstrated good repeatability, with no significant difference in the maximum lactate to total carbon signal (Lac_max_) between injections at two time points. There was a significantly higher Lac_max_ in the tumours compared with the normal gland, and lesion detection was increased using hyperpolarised ^13^C-MRI compared with ^1^H-MRI.^42^

**Fig. 3:**
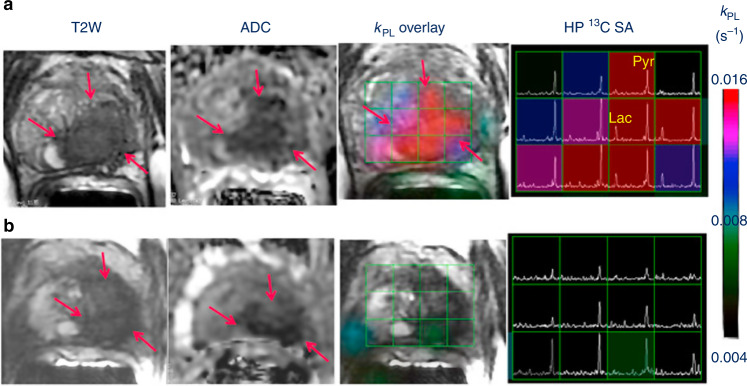
Hyperpolarised ^13^C-MRI in a patient with metastatic prostate cancer. Hyperpolarised ^13^C-MRI in a patient with metastatic prostate cancer undergoing androgen deprivation therapy before and after 6 weeks of treatment initiation.^69^ Representative axial T2-weighted (T2W) anatomic image and corresponding apparent diffusion coefficient (ADC) image, T2W image with an overlaid pyruvate-to-lactate metabolic exchange rate (*k*_PL_) image and corresponding hyperpolarised ^13^C spectral array are shown. The 52-year-old prostate cancer patient with extensive high-grade prostate cancer was imaged **a** before therapy and **b** 6 weeks after initiation of androgen ablation and chemotherapy. Before treatment, the region of prostate cancer can be clearly seen (red arrows) as a reduction in signal on the T2W and ADC images, and increased hyperpolarised lactate and associated *k*_PL_ on hyperpolarised ^13^C-MRI. After initiation of androgen deprivation therapy there was a significant reduction in hyperpolarised lactate and *k*_PL_ to normal levels, with the prostate volume and ADC showing only a modest response to treatment. Reprinted from European Urology, volume 72, Aggarwal, R., Vigneron, D. B. & Kurhanewicz, J., Hyperpolarized 1-[13C]-pyruvate magnetic resonance imaging detects an early metabolic response to androgen ablation therapy in prostate cancer, pages 1028–1029, Copyright (2017), with permission from Elsevier.

Furthermore, a separate report demonstrated the first clinical case of using the technique for response assessment in a patient with metastatic prostate cancer and a serum PSA level of 25.2 ng/ml (Fig. 3).^69^ After 6 weeks’ treatment with antiandrogen and chemotherapy, hyperpolarised ^13^C-MRI of the prostate revealed a demonstrable metabolic response, with a return of the lactate signal-to-noise ratio to normal levels; by contrast, tumour volume on T2-weighted images on MRI decreased only moderately and the apparent diffusion coefficient (ADC) on diffusion-weighted imaging (DWI) showed only a slight increase, indicating only a modest change in tumour cellularity using this approach. The positive treatment response was clinically confirmed 6 months later, when serum PSA levels had decreased and were no longer measurable.

### Renal cell carcinoma

The number of early-stage renal cancers that are being detected is increasing, partly due to the rise in the use of cross-sectional imaging such as CT and MRI.^86^ However, despite the early detection and subsequent treatment of these cancers, there has been no decrease in disease-specific mortality, which suggests that a relevant proportion of these patients might receive overtreatment. Clear cell renal cell carcinoma (ccRCC) has many features that render it amenable to assessment with hyperpolarised ^13^C-MRI: it is highly glycolytic but well perfused, which is ideal for imaging using an intravenous metabolic contrast agent with a short signal lifetime; it is morphologically and genetically heterogeneous, thus providing a model of metabolic heterogeneity; there is increasing evidence that RCC is driven by metabolic dysregulation, and some renal tumours arise from mutations in mitochondrial enzymes, making it an ideal model to study novel tumour metabolism; and alternative methods to image metabolism, such as [^18^F]FDG-PET, are limited by contamination from renal excretion of the tracer. Furthermore, as RCC has a poor prognosis, both new treatments and imaging methods to monitor response are required.

Hyperpolarised ^13^C-MRI has the potential to improve treatment selection in patients with ccRCC by noninvasively predicting tumour aggressiveness. This hypothesis is supported by evidence from studies demonstrating that the expression of *LDHA*, which encodes a subunit of LDH, correlated positively with tumour grade and negatively with progression-free survival and overall survival.^87^ As hyperpolarised pyruvate and^13^C-MRI assesses a reaction catalysed by the enzyme LDH, the imaged lactate is likely to correlate with tumour grade and outcome as well. Noninvasive prediction of tumour aggressiveness and patient stratification using current state-of-the-art imaging techniques has poor accuracy, and invasive diagnostic approaches suffer from low sensitivity: biopsy samples have been shown to frequently undergrade RCCs due to the high degree of intratumoural heterogeneity, and up to eight biopsy samples are required to fully evaluate driver mutational status.^88^ Hyperpolarised ^13^C-MRI might therefore also provide a powerful tool to target tissue sampling and to provide a metabolic readout to be used in conjunction with the biopsied tissue to minimise undersampling and undergrading.

Preliminary case reports on the use of dynamic hyperpolarised ^13^C-MRI in ccRCC have shown promise.^55,89^ The results have demonstrated spatial variation in the lactate signal across these tumours, which corresponds to the ex vivo measurements of tissue lactate: for example, in multiple tumour biopsy samples obtained both intraoperatively and from the nephrectomy specimens of patients who had previously undergone hyperpolarised ^13^C-MRI of the tumour, the tissue lactate concentration measured using liquid-chromatography mass-spectrometry (LCMS) correlated significantly with the lactate signal-to-noise ratio using hyperpolarised ^13^C-MRI, both in intraoperative and post-nephrectomy samples, thus demonstrating biologic validity of the method for the first time in humans.^55,89^

### Breast cancer

Neoadjuvant chemotherapy is increasingly gaining importance in the treatment of early breast cancer and is an important clinical area in which hyperpolarised ^13^C-MRI could be used as a tool for early response assessment, as well as having a potentially significant impact on clinical decision making. A 2018 meta-analysis has demonstrated that neoadjuvant chemotherapy increases breast-conservation rates without compromising on distant recurrence, breast cancer survival or overall survival (OS).^90^ Pathological complete response (pCR) after neoadjuvant chemotherapy has been shown to predict recurrence-free survival (RFS), event-free survival (EFS) and OS, especially in patients with HER2^+^ breast cancer and triple-negative breast cancer (TNBC).^91,92^ An additional benefit of neoadjuvant chemotherapy is that it allows any response to therapy to be assessed in vivo, while the tumour is still measurable: the clinical response assessment is currently largely based on monitoring the maximum tumour diameter using routine clinical radiological examinations.^93^

However, the prediction of successful response to neoadjuvant chemotherapy is still a significant clinical challenge. At present MRI is the most accurate imaging modality for the assessment of tumour response to neoadjuvant therapy,^94^ and several meta-analyses indicate pooled sensitivities for the identification of residual tumour after completion of preoperative therapy in patients, that range between 83% and 88% and pooled specificities of 54–83%.^95,96^ As in the case of mpMRI of the prostate, additional approaches are required to improve sensitivity and specificity. DWI MRI and [^18^F]FDG-PET have shown promise in predicting pCR at earlier time points during neoadjuvant chemotherapy. Identifying nonresponders at this early timepoint would be highly desirable to allow their management to be changed (for example, to early surgery) in order to minimise the risk of metastases from chemoresistant cells and to reduce the side effects of prolonged but ineffective chemotherapy. It has been demonstrated that the addition of the ADC measured with DWI to standard functional tumour volume assessed on contrast-enhanced MRI showed improvement in the prediction of treatment response in hormone receptor positive (HR^+^) and TNBC, increasing the overall AUC from 0.76 to 0.81 at post-neoadjuvant chemotherapy.^97^ When [^18^F]FDG-PET is performed in conjunction with MRI, the sensitivity and specificity have reached up to 100% and 71%, respectively, after only one cycle of chemotherapy.^98^ By contrast, 3ʹ-deoxy-3ʹ-[^18^F]-fluorothymidine (FLT) PET has been shown to only weakly predict pCR after one cycle of neoadjuvant chemotherapy.^99^ Despite initial promising results for a role for ^1^H-MRS in monitoring neoadjuvant chemotherapy, in which an early decrease in tCho indicated a good response to therapy between 24 h after the initial dose and after 1–2 cycles,^22,100^ it has only shown limited predictive power for pCR or radiological response.^32^ ^1^H-MRS is also technically challenging in the setting of a multicentre trial, partly due to long acquisition times and limited technical support on clinical scanners and picture archiving and communication systems (PACS).

New imaging methods are therefore required to predict the response to therapy sooner and more accurately, which would be desirable for clinical decision making. Such new methods are particularly important for combinational and targeted drug regimens used clinically, and also as companion biomarkers within clinical trials investigating novel targeted agents such as poly-ADP-ribosyl polymerase (PARP) inhibitors, phosphoinositide 3-kinase (PI3K) pathway inhibitors, mTOR inhibitors such as everolimus, immune checkpoint inhibitors and cyclin-dependent kinase (CDK) inhibitors.

Metabolic readouts could enable a very early assessment of successful response to therapy, given the downstream convergence of these pathways towards alterations in glycolytic reactions,^101^ making hyperpolarised ^13^C-MRI a promising tool to study metabolic changes in response to therapy. Preclinical studies have demonstrated that a decrease in label exchange between pyruvate and lactate can be detected in animal models of breast cancer as soon as 24 h after initiation of successful treatment^67^ and have provided evidence that hyperpolarised ^13^C-MRI might be more sensitive to very early-treatment-related metabolic changes than [^18^F]FDG-PET.^14^

**Fig. 4:**
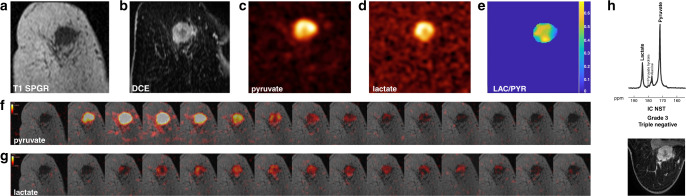
Hyperpolarised ^13^C-MRI in a case of triple-negative breast cancer (TNBC) **a** Coronal T1 3D spoiled gradient echo (SPGR) MRI image. **b** Coronally reformatted DCE image at peak enhancement after intravenous injection of a gadolinium-based contrast agent. **c** Summed hyperpolarised ^13^C-pyruvate and **d** summed hyperpolarised ^13^C-lactate images. The area of low ^13^C-pyruvate and ^13^C-lactate signals in the centre of the tumour are likely to correspond to an area with low enhancement on DCE. **e** LAC/PYR map showing intratumoural heterogeneity. The dominant intratumoural heterogeneity was concordant between the DCE-MRI and hyperpolarised ^13^C-MRI images and represents decreased delivery of both the gadolinium-based contrast agent and ^13^C-pyruvate to the centre of the tumour. **f**, **g** Dynamic hyperpolarised ^13^C-pyruvate and ^13^C-lactate images acquired over 15 time points after intravenous injection of hyperpolarised [1-^13^C]pyruvate (delay = 12 seconds; temporal resolution = 4 seconds). **h** Top: ^13^C metabolite spectrum from a coronal dynamic IDEAL spiral CSI slice covering the tumour summed over 15 time points; Bottom: The axial image from the equivalent DCE-MRI data was taken at the timepoint of maximum tumour enhancement. ppm parts per million, IC NST invasive cancer of no specific type. This Figure was reproduced from^43^ (10.1073/pnas.1913841117), licenced under CC-BY-NC-ND (https://creativecommons.org/licenses/by-nc-nd/4.0/).

A first clinical hyperpolarised ^13^C-MRI study in patients with early breast cancer demonstrated varying levels of lactate signal-to-noise ratio and lactate-to-pyruvate ratio across different tumour subtypes. Higher lactate signal-to-noise ratio was observed in the more aggressive and TNBC tumours, which makes these tumours potential candidates for early response assessment^43^ (Fig. 4). These more aggressive tumours are more likely to undergo neoadjuvant chemotherapy, thus supporting the potential use of hyperpolarised ^13^C-MRI in this context. The metabolic intratumoural heterogeneity observed in TNBC using hyperpolarised ^13^C-MRI could make this approach a potential prognostic imaging biomarker, as several genomic studies have demonstrated that high levels of intratumoural heterogeneity are associated with poor prognosis, which might be due to a decreased immune response.^102^ The clinical hyperpolarised ^13^C-MRI study showed a significant correlation between the lactate-to-pyruvate ratio and the levels of MCT1, thus providing a biochemical mechanism for the variation in lactate labelling in breast cancer.^43^ Furthermore, the lactate-to-pyruvate ratio also correlated with tumour volume and the expression of hypoxia-inducible factor (HIF)-1α, suggesting that hypoxia might account for the increased lactate labelling in larger breast tumours.

The first report of assessing treatment response with hyperpolarised ^13^C-MRI in a patient with TNBC showed that an early response to neoadjuvant chemotherapy could be demonstrated as a 34% decrease in ^13^C-lactate labelling after a single treatment cycle, whereas dynamic contrast-enhanced MRI (DCE-MRI) and pharmacokinetic modelling did not clearly identify treatment response at this timepoint.^56^ Future larger studies are required to more fully assess the role of the technique in this context.

### Pancreatic cancer

Pancreatic ductal adenocarcinoma (PDAC) is characterised by high morbidity, with 5-year survival rates as low as 10%. More than 50% of patients are diagnosed with advanced pancreatic cancer and undergo palliative treatment while only 10–20% are diagnosed with early-stage disease that is suitable for potentially curative surgery.^103^ The pancreatic tumour niche is characterised by high interstitial pressure, dense fibrotic stroma, low tumour cellularity, and reduced vasculature.^104,105^ To overcome local nutrient sparsity, PDAC engages in metabolic crosstalk, with tumour cells deriving some of their nutrients from other intratumoural cell populations.^106^ For example, lactate might be secreted by hypoxic tumour cells and taken up by cancer cells in normoxic portions of the tumour to fuel proliferation.^107^

**Fig. 5:**
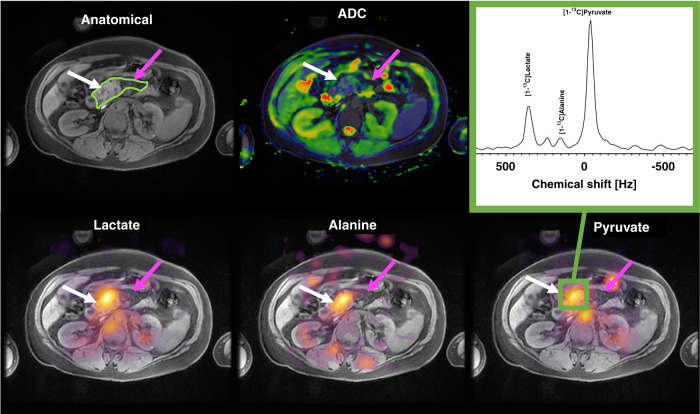
Hyperpolarised ^13^C-MRI on a patient with pancreatic cancer. Top row: anatomical axial slice showing the pancreatic tumour (white arrow) and the exocrine pancreas (purple arrow); the green region of interest outlines the whole pancreas. The ADC is greatly reduced in the tumour tissue. Bottom row: high [1‐^13^C]pyruvate, [1‐^13^C]lactate and [1‐^13^C]alanine signals were observed in the pancreatic tumour, with images acquired >30 sec after injection of hyperpolarised [1‐^13^C]pyruvate. The spectrum acquired in the area of the pancreatic cancer shows [1‐^13^C]pyruvate, [1‐^13^C]lactate and [1‐^13^C]alanine peaks. Reprinted from^50^ with permission from John Wiley & Sons.

Hyperpolarised ^13^C-MRI has the potential to improve diagnostic accuracy when characterising lesions that are incidentally detected on imaging and could guide management in patients undergoing neoadjuvant chemotherapy in a similar way to that described for breast cancer. In a preclinical study in murine models of pancreatic preneoplasia and invasive cancers, hyperpolarised ^13^C-MRI was been shown to detect preneoplasia based on an altered alanine-to-lactate ratio and was able to distinguish between invasive and noninvasive lesions based on this semiquantitative parameter.^49^ In a first clinical report of two patients with advanced pancreatic cancer who had undergone several cycles of chemotherapy, the pancreatic adenocarcinoma could still be distinguished from the adjacent normal tissue based on elevated lactate levels due to increased levels of glycolysis and elevated alanine levels (Fig. 5).^50^ The increase in the levels of alanine might be due to its secretion by exocrine stellate cells as a nutrient for adenocarcinoma cells in a hypoxic and nutrient-deprived tumour environment.^108,109^

## Conclusions and future perspectives

Results from preclinical studies indicate that hyperpolarised ^13^C-MRI has a number of potential clinical applications: tumour characterisation based on their metabolic profile; noninvasive tumour grading; targeting of the most aggressive tumour regions in the presence of heterogeneity and assessment of treatment response sooner than is attainable using current state-of-the-art imaging methods. For hyperpolarised ^13^C-MRI to transition from a research tool to a robust and reliable clinical imaging method for probing cancer metabolism, technical and biological validation of the procedure is required. Quantitative and semiquantitative metrics of hyperpolarised ^13^C-pyruvate metabolism need to be correlated with invasive results from tissue analysis to understand the biological drivers for the imaging measurements. For example, comparing the hyperpolarised ^13^C-lactate signal to the endogenous lactate pool and the expression of MCTs or LDH will help to reveal the biochemical mechanisms involved in different tumour types: i.e. to determine whether membrane transport of the ^13^C-labelled pyruvate or enzyme mediated ^13^C-label exchange between pyruvate and lactate is the dominant factor in each case. Comparison of hyperpolarised ^13^C-MRI with state-of-the-art imaging for cancer detection will help to establish the added value of imaging metabolism over conventional anatomical and functional imaging methods. Repeatability and reproducibility studies are required to understand how variable the technique is and the magnitude of the change in tissue metabolism that is required for detection. Furthermore, harmonisation of acquisition and analysis methods will aid multicentre trials that are required to establish the clinical role of the technique.^60,61^

The field of clinical hyperpolarised ^13^C-MRI faces many challenges: the complexity of the clinical kits used for injectable hyperpolarised pyruvate solutions; the requirements for determining quality assurance for human use; optimising acquisition, analysis and quantification of dynamic metabolite measurements due to rapid signal decay; inherent low spatial resolution; as well as requirements for adequate repeatability and reproducibility. To accomplish full translation and integration into clinical cancer imaging, these challenges need to be overcome and hyperpolarised ^13^C-MRI needs to be integrated into clinical trials alongside state-of-the art imaging so that both, ^13^C-MRI and conventional imaging can be directly compared with clinical outcome measures such as response and survival. These trial designs will allow not only allow the clinical utility of the technique to be evaluated, but also the potential to affect treatment decisions and patient management. The next step will be to incorporate hyperpolarised ^13^C-MRI into treatment decision-making endpoints within these trials; positive results from such studies would allow full integration into clinical care.^59^ The area of cancer imaging where the evidence for this translation is most promising is the assessment of early response; however, the ideal timepoint for assessing these metabolic endpoints needs to be identified, such that it is early enough to provide clinical advantage over conventional imaging but late enough to reliably and specifically identify nonresponders. Future multicentre trials of the technology will address these important clinical questions.

## Data Availability

Not applicable
